# Oxidative stress-induced chromosome breaks within the *ABL* gene: a model for chromosome rearrangement in nasopharyngeal carcinoma

**DOI:** 10.1186/s40246-018-0160-8

**Published:** 2018-06-18

**Authors:** Sang-Nee Tan, Sai-Peng Sim, Alan Soo-Beng Khoo

**Affiliations:** 1 Department of Paraclinical Sciences, Faculty of Medicine and Health Sciences, Universiti Malaysia Sarawak, Sarawak, Malaysia; 2 Molecular Pathology Unit, Cancer Research Centre, Institute for Medical Research, Kuala Lumpur, Malaysia

**Keywords:** NPC, Oxidative stress, H_2_O_2_, Apoptosis, *ABL*, MAR/SAR

## Abstract

**Background:**

The mechanism underlying chromosome rearrangement in nasopharyngeal carcinoma (NPC) remains elusive. It is known that most of the aetiological factors of NPC trigger oxidative stress. Oxidative stress is a potent apoptotic inducer. During apoptosis, chromatin cleavage and DNA fragmentation occur. However, cells may undergo DNA repair and survive apoptosis. Non-homologous end joining (NHEJ) pathway has been known as the primary DNA repair system in human cells. The NHEJ process may repair DNA ends without any homology, although region of microhomology (a few nucleotides) is usually utilised by this DNA repair system. Cells that evade apoptosis via erroneous DNA repair may carry chromosomal aberration. Apoptotic nuclease was found to be associated with nuclear matrix during apoptosis. Matrix association region/scaffold attachment region (MAR/SAR) is the binding site of the chromosomal DNA loop structure to the nuclear matrix. When apoptotic nuclease is associated with nuclear matrix during apoptosis, it potentially cleaves at MAR/SAR. Cells that survive apoptosis via compromised DNA repair may carry chromosome rearrangement contributing to NPC tumourigenesis. The Abelson murine leukaemia (*ABL)* gene at 9q34 was targeted in this study as 9q34 is a common region of loss in NPC. This study aimed to identify the chromosome breakages and/or rearrangements in the *ABL* gene in cells undergoing oxidative stress-induced apoptosis.

**Results:**

In the present study, in silico prediction of MAR/SAR was performed in the *ABL* gene. More than 80% of the predicted MAR/SAR sites are closely associated with previously reported patient breakpoint cluster regions (BCR). By using inverse polymerase chain reaction (IPCR), we demonstrated that hydrogen peroxide (H_2_O_2_)-induced apoptosis in normal nasopharyngeal epithelial and NPC cells led to chromosomal breakages within the *ABL* BCR that contains a MAR/SAR. Intriguingly, we detected two translocations in H_2_O_2_-treated cells. Region of microhomology was found at the translocation junctions. This observation is consistent with the operation of microhomology-mediated NHEJ.

**Conclusions:**

Our findings suggested that oxidative stress-induced apoptosis may participate in chromosome rearrangements of NPC. A revised model for oxidative stress-induced apoptosis mediating chromosome rearrangement in NPC is proposed.

**Electronic supplementary material:**

The online version of this article (10.1186/s40246-018-0160-8) contains supplementary material, which is available to authorized users.

## Background

Nasopharyngeal carcinoma (NPC) is a malignant neoplasm derived from mucosal epithelium of the nasopharynx. According to the World Health Organization (WHO), NPC can be classified into three subtypes according to the degree of epithelial differentiation, namely keratinising squamous cell carcinoma (Type I), non-keratinising squamous cell carcinoma (Type II) and undifferentiated or poorly differentiated carcinoma (Type III) [1].

NPC is a rare malignancy in most parts of the world; the incidence rates are below one per 100,000 persons per year [2, 3]. However, there are a few well-known notable exceptions [3]. The intermediate rates were reported in South-Eastern Asia, Northern Africa, the Middle East and Arctic Region [3, 4]. The highest incidence rate was observed among Southern Chinese living in central Guangdong province. The annual incidence rates for males and females in central Guangdong province are 23.3 per 100,000 and 8.9 per 100,000, respectively [4]. The NPC incidence rates are generally increasing from Northern China to Southern China [3, 4]. In addition, an exceptionally high incidence rate has been reported among the Bidayuh people, the second biggest ethnic group in Sarawak, Malaysia. The age-adjusted rate of Sarawak residents is 13.5 per 100,000 and 6.2 per 100,000 in males and females, respectively. Although the average rate in Sarawak is intermediate, the incidence rate for Bidayuh people is about 50% higher than that in Hong Kong (a part of the Cantonese region of Guangdong province) [5].

NPC is strongly associated with Epstein-Barr Virus (EBV) infection [6–8] as well as dietary [9–11], environmental [12] and genetic factors [13, 14]. Several genetic aberrations have been reported to be related to the development of NPC, suggesting that NPC tumourigenesis involves multiple genetic changes. These include chromosomal gains or losses [15–19], loss of heterozygosity (LOH) [20–23], homozygous deletions [24–27], promoter hypermethylation of tumour suppressor genes [28–31] and shortening of chromosome telomeres [32, 33].

Although the consistent chromosome rearrangements have long been identified in NPC, the molecular mechanism underlying the chromosome rearrangements of NPC remains poorly understood. In addition to EBV infection, long-term exposures to nitrosamines, formaldehyde, cigarette smoke and wood dust have all been found to be associated with NPC [12, 34–36]. More recently, much concern has been raised about the association between chronic inflammation of sinonasal tract and NPC [37, 38]. It is remarkable that all these aetiological factors may trigger oxidative stress [39–43]. Oxidative stress is an imbalance of pro-oxidants and antioxidants resulting in a disruption of redox signalling and control. Pro-oxidants induce oxidative stress either through excessive production of reactive oxygen species (ROS) or inhibition of antioxidant systems [44]. ROS are chemically-reactive molecules containing oxygen which include peroxyl RO·, hydroxyl radical OH·, superoxide O^2^·- and hydrogen peroxide H_2_O_2_ [45]. ROS cause several kinds of DNA damages, including strand cleavage, base modification and DNA-protein cross-linkage [45, 46]. Importantly, formaldehyde and acrolein, a component of cigarette smoke, are reactive aldehydes. In addition, reactive aldehydes may also be produced endogenously during oxidative stress. Aldehydes may cause adduct formation that impairs the function of DNA, RNA and proteins via electrophile-nucleophile interaction. Exposure to environmental aldehydes has been shown to be associated with the onset and development of human diseases that involve oxidative stress. It has been suggested that environmental and endogenous aldehydes may interact additively and exacerbate the cellular oxidative damage [47].

An evaluation of the levels of 8-hydroxy-2′-deoxyguanosine (8-OHdG), a biomarker of oxidative DNA damage, had been done among NPC patients. The tissue and serum levels of 8-OHdG in NPC patients have been found to be significantly higher than those in control patients [48]. Oxidative stress was suggested to play an important role in carcinogenesis [49]. Since there is a strong link between the aetiological factors of NPC and oxidative stress, it is intriguing to investigate the role of oxidative stress in the molecular mechanisms underlying chromosome rearrangements of NPC.

Oxidative stress may induce apoptosis [50, 51]. H_2_O_2_ has been well known as an apoptotic inducer for various human cell types, including osteoblasts [52, 53], sarcoma cells [54], osteosarcoma cells [55], hepatoma cells [56], astrocytoma cells [57], Jurkat T lymphocytes [58] and Fanconi’s anaemia cells [59]. Apoptosis or programmed cell death was first described by Kerr et al. (1972). Apoptosis is a type of genetically controlled cell suicide which occurs naturally in multicellular organisms in order to eliminate poisonous cells. Apoptosis is morphologically characterised by condensation of chromatin, fragmentation of nuclei, compaction of cytoplasmic organelles, cell shrinkage and cytoplasmic membrane blebbing [60–62]. Apoptosis is related to several biochemical events, including externalisation of phosphatidylserine (PS) on cell membrane, alteration in mitochondrial membrane potential (MMP), release of cytochrome *c* (cyt *c*) from mitochondria, caspase activation and internucleosomal cleavage of DNA [63].

The alteration of nuclear chromatin during apoptosis is often associated with fragmentation of the genomic DNA into high-molecular-weight (HMW) DNA of 30 to 50 and 200 to 300 kbp [64, 65]. These sizes of fragments have been suggested to be derived from the release of loops (50 kbp) or rosettes (300 kbp) of chromatin, probably when they become detached from their binding sites on the nuclear scaffold [66]. Further degradation of the HMW DNA produces the internucleosomal DNA fragments of 180 to 200 bp [67, 68].

Cells undergoing apoptosis may recover from the execution phase of apoptosis upon DNA repair [69, 70]. There are two major double-strand breaks (DSBs) repair pathways, namely homologous recombination (HR) and non-homologous end joining (NHEJ) [71, 72]. Chromosomal DSB repair by HR is predominant during late S/G2 phases of the cell cycle. NHEJ is the more frequently used pathway that can repair a DSB at any time during the cell cycle. These two pathways have different degree of requirement for DNA homology. The HR pathway requires sufficient homology, usually more than 100 bp. Given that the HR DNA repair system ligates two DNA ends with homologous sequences, it gives rise to precise DNA repair. The NHEJ DNA repair system joins two DNA ends without intensive requirement of sequence homology. This pathway joins two DNA terminals with microhomology of a few base pairs [71, 73, 74]. NHEJ pathway has been shown to be prone to cause erroneous repair of DSBs. This may in turn lead to chromosomal aberrations [75]. It has been suggested that interaction of the NHEJ DNA repair system with apoptosis can act as a mechanism leading to translocation in leukaemia [70].

Chromosomal breakage takes place in the initial stage of chromosome rearrangement and apoptotic DNA fragmentation. It has been observed that chromosome breaks do not randomly occur throughout a gene. Rather, chromosome breaks normally fall within certain regions that contain specific chromatin structures, such as matrix association region/scaffold attachment region (MAR/SAR) [76, 77]. MAR/SAR are DNA sequences where DNA loop structure attaches to nuclear scaffold/matrix proteins [78]. There are two breakpoint cluster regions (BCR) identified in the *AF9* gene. These two BCRs are bordered by two experimentally isolated MAR/SARs [76]. The BCR of the mixed lineage leukaemia (*MLL*) gene has also been found to contain two MAR/SAR sequences [78]. In addition, the most crucial apoptotic nuclease CAD has been reported to associate with the nuclear matrix of apoptotic cells [79].

We previously demonstrated that in normal nasopharyngeal epithelial and NPC cells, oxidative stress-induced apoptosis resulted in chromosome breaks in the *AF9* gene located on chromosome 9p22. We further demonstrated that caspase-activated DNase (CAD) may be a major player in mediating the oxidative stress-induced chromosomal cleavages. A few chromosome breaks were identified within the *AF9* region that was previously reported to participate in translocation in an acute lymphoblastic leukaemia (ALL) patient. These findings suggested that oxidative stress-induced apoptosis may play an important role in mediating chromosome rearrangements in NPC [80]. In the present study, we further investigated the potential role of oxidative stress-induced apoptosis by targeting the Abelson murine leukaemia viral oncogene homologue 1 (*ABL*) gene located on chromosome 9q34. This study targeted the *ABL* gene because 9q34 is a common region of loss in NPC [23].

The *ABL* gene is a proto-oncogene which encodes a 150 kDa nonreceptor protein tyrosine kinase. It was first recognised as the cellular homologue of the *v-abl* oncogene product of the Abelson murine leukaemia virus [81, 82]. The ABL protein has a complex structure that contains many domains. These domains are found in proteins which are involved in the formation of complexes in signal transduction pathway. It has been demonstrated that overexpression of *ABL* in fibroblast resulted in growth arrest [83]. The product of *ABL*-*BCR* fusion appears to be an abnormal kinase that stimulates the proliferation of myeloid cells leading to chronic myelogenous leukaemia (CML) [84]. The *ABL* gene is 173,795 bp in length and it consists of 11 exons [Ensembl:ENSG00000097007]. The description of exons and introns in the *ABL* gene is shown in Additional file 1.

By using MAR/SAR recognition signature (MRS), we predicted 12 possible MAR/SAR sites in the *ABL* gene. We demonstrated that oxidative stress-induced apoptosis resulted in chromosome breaks in the *ABL* BCR which contains a MAR/SAR site. We detected shift translocations in H_2_O_2_-treated normal nasopharyngeal epithelial cells. Interestingly, we found region of microhomology at the breakpoint junctions. This observation suggests a role for NHEJ DNA repair system in mediating the translocation. At last, we illustrated the possible role of oxidative stress-induced apoptosis in mediating chromosome rearrangements in NPC via NHEJ DNA repair system.

## Results

### In silico prediction of MAR/SAR by using MAR/SAR recognition signature (MRS)

Potential MAR/SAR sites in the *ABL* gene were predicted by using MRS. MRS is a bipartite sequence that is strongly associated with MAR/SAR [85]. This bipartite sequence consists of 16 bp nucleotide motif (AWWRTAANNWWGNNNC) within a distance of 200 bp of the 8 bp nucleotide motif (AATAAYAA). However, for our preliminary results in the *ABL* gene, we only found one MRS (MAR/SAR 9 in Table 1) in the biochemically identified SAR1 [77]. The distance between the 8 bp sequence element and the 16 bp sequence element was 248 bp. Therefore, in this study, we set the maximal distance between the two sequence elements at 250 bp.

**Table 1:** MAR/SAR predicted in the *ABL* gene

Predicted MAR/SAR	AWWRTAANNWWGNNNC (16 bp)	Nucleotide position	AATAAYAA (8 bp)	Nucleotide position	Distance (bp)	Location in exon/intron
1–1	ATTGTAACCATATCTC (C)	26,055–26,070	AATAATAA (C)	26,118–26,125	+ 47	Intron 1
1–2	ATCATAACTTAGCAAC (C)	26,613–26,628	AATAACAA (W)	26,565–26,572	−40	Intron 1
2	AAAAAAATTTTGTACC (C)	29,601–29,616	AATAATAA (C)	29,629–29,636	+ 12	Intron 1
3–1	ATAATAATTATACAAC (C)	80,698–80,713	AATAATAA (W)	80,726–80,733	+ 12	Intron 1
	AATATAAATAAAGTGC (W)	80,732–80,747			Overlap	
3–2	ATTGTAACTAAGGTTC (C)	81,458–81,473	AATAACAA (C)	81,422–81,429	−28	Intron 1
4	ATAATAATAAAGAGAT (W)	99,259–99,274	AATAATAA (W)	99,261–99,268	Overlap	Intron 1
	AATATAATCAACTGAC (W)	99,447–99,462			− 178	
5	ATAAAAAGGAAGATTC (W)	105,498–105,513	AATAATAA (C)	105,616–105,623	+ 102	Intron 1
6	AAAAAAAAAAAGACTC (C)	108,315–108,330	AATAATAA (C)	108,484–108,491	+ 153	Intron 1
			AATAATAA (C)	108,487–108,494	+ 156	
			AATAATAA (C)	108,490–108,497	+ 159	
7	AATGTAACAGAGAGCC (C)	117,122–117,137	AATAACAA (W)	117,343–117,350	+ 205	Intron 1
8	AAAATAAACATATACC (W)	119,757–119,772	AATAATAA (W)	119,739–119,746	− 10	Intron 1
9	AAAGTAAAATTGAAAG (C)	133,546–133,561	AATAACAA (W)	133,810–133,817	+ 248	Intron 1
10	ATTACAAGTTTGGTAC (C)	144,212–144,227	AATAATAA (C)	143,996–144,003	− 208	Intron 3
11	ATAAAAACAAAGAAGC (C)	163,018–163,033	AATAACAA (W)	163,048–163,055	+ 14	Intron 7
12	AAAATAATAATGGCCA (W)	167,856–167,871	AATAATAA (W)	167,858–167,865	Overlap	Intron 10

Nucleotide positions of the MRSs with their sequence composition, relative orientation (C, Crick strand and W, Watson strand), distance between the two sequence elements and location of the MRSs in the exon or intron of the *ABL* gene are shown. A negative distance indicates that 8 bp sequence element precedes the 16 bp sequence element

**Fig. 1:**
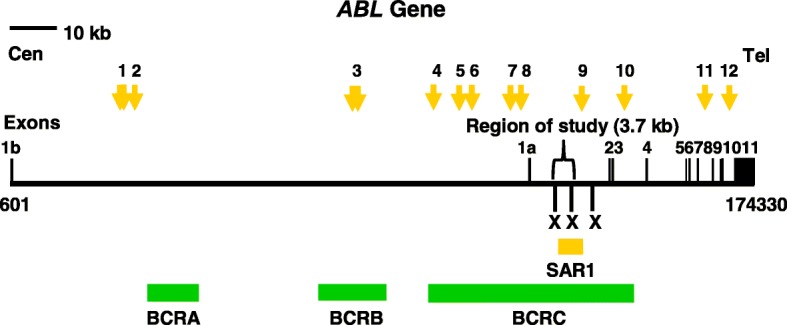
Distribution of potential MAR/SAR sites predicted in the *ABL* gene. The *ABL* genomic map from nucleotide positions 601-174330 is illustrated above [Ensembl:ENSG00000097007]. The locations of exons 1 to 11 are shown. Green boxes represent the three previously reported patient breakpoints cluster regions which are designated as BCRA, BCRB and BCRC. Yellow box shows the previously biochemically extracted MAR/SAR which is designated as SAR1 [77]. Yellow arrows represent the potential MAR/SARs predicted by MRS. Clusters of more than one MRS within close proximity are regarded as a single potential MAR/SAR site. For instance, there were two MRSs predicted in BCRB, however, they were regarded as a single potential MAR/SAR site (MAR/SAR 3) because they were found in close proximity. There was one MAR/SAR site (MAR/SAR 9) predicted in the experimentally isolated SAR1

By using MRS, we predicted 12 potential MAR/SAR sites in the *ABL* gene. The nucleotide positions of the MRSs with their sequence composition, relative orientation, distance between the two sequence elements and location of the MRSs in the exon or intron of the *ABL* gene are shown in Table 1. Out of the 12 predicted MAR/SAR sites, 9 were identified in intron 1 which is the largest intron (approximately 140 kb in length) in the *ABL* gene (approximately 175 kb in length) (MAR/SAR 1–9 in Table 1). One potential MAR/SAR site was separately found in intron 3 (MAR/SAR 10 in Table 1) and intron 10 (MAR/SAR 11 in Table 1). The distribution of the predicted MAR/SAR sites in the *ABL* gene is shown in Fig. 1. One MAR/SAR site (MAR/SAR 9 in Table 1) was predicted within the biochemically defined SAR1 which is located in the second intron 1 [77].

### Apoptosis detection

NP69 cells were either left untreated or treated with 100 μM of H_2_O_2_ for 16 and 24 h while HK1 cells were either left untreated or treated with 50 μM for 4 and 8 h. Cells treated with CPT was included as a positive control. The cells were then subjected to flow cytometric analyses of PS externalisation and MMP loss.

### Phosphatidylserine (PS) externalisation

**Fig. 2:**
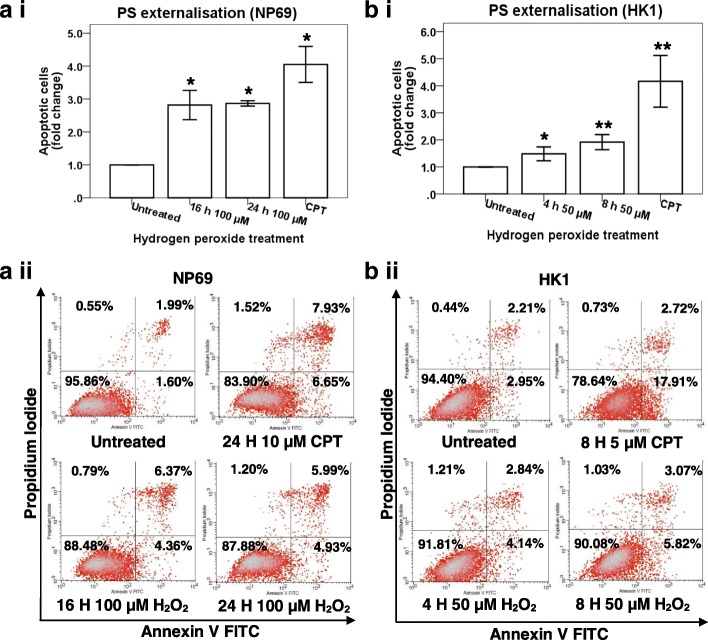
Flow cytometric analysis of phosphatidylserine (PS) externalisation. NP69 cells were either left untreated or treated with 100 μM of H_2_O_2_ for 16 and 24 h while HK1 cells were either left untreated or treated with 50 μM for 4 and 8 h. Cells treated with CPT was included as a positive control. The percentage of cells showing PS externalisation was determined in H_2_O_2_-treated NP69 cells (**a i**) and HK1 cells (**b i**). Means and SD of three independent experiments performed in duplicate are shown. Data are expressed as fold change normalised to untreated control. *p < 0.01, **p < 0.001 (Student’s *t* test). The representative dot plot diagrams indicating the apoptotic populations of (**a ii**) H_2_O_2_-treated NP69 cells and (**b ii**) H_2_O_2_-treated HK1 cells are shown. The lower left quadrants indicate healthy cells; the lower right quadrants indicate cells in early apoptosis; the upper right quadrants indicate cells in late apoptosis and necrosis

As shown in Fig. 2a i, the percentages of apoptotic cells detected in NP69 treated with 100 μM of H_2_O_2_ for 16 and 24 h were 2.82-fold (*p* = 0.000170) and 2.87-fold (*p* = 3.4346E−8) higher than that detected in the untreated control, respectively. The percentages of apoptotic cells detected in HK1 treated with 50 μM of H_2_O_2_ for 4 and 8 h were 1.48-fold (*p* = 0.005735) and 1.92-fold (*p* = 0.000477) higher than that detected in the untreated control, respectively (Fig. 2b i). Figure 2a i and b ii are the representative dot plot diagrams showing the apoptotic population of H_2_O_2_-treated NP69 and HK1 cells, respectively.

### Mitochondrial membrane potential (MMP) loss

**Fig. 3:**
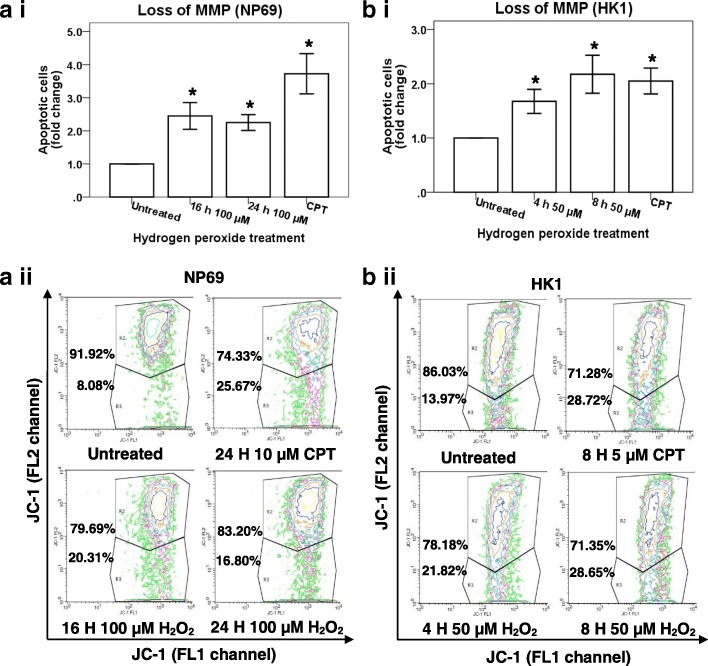
Flow cytometric analysis of mitochondrial membrane potential (MMP) loss. NP69 cells were either left untreated or treated with 100 μM of H_2_O_2_ for 16 and 24 h while HK1 cells were either left untreated or treated with 50 μM for 4 and 8 h. Cells treated with CPT was included as a positive control. The percentage of cells showing MMP loss was determined in H_2_O_2_-treated NP69 cells (**a i**) and HK1 cells (**b i**). Means and SD of two independent experiments performed in duplicate are shown. Data are expressed as fold change normalised to untreated control. *p < 0.01 (Student’s *t* test). The representative contour plot diagrams indicating the apoptotic populations of (**a ii**) H_2_O_2_-treated NP69 cells and (**b ii**) H_2_O_2_-treated HK1 cells are shown. The upper quadrants indicate healthy cells whereas the lower quadrants indicate cells expressing MMP loss

As shown in Fig. 3a i, the percentages of apoptotic cells detected in NP69 treated with 100 μM of H_2_O_2_ for 16 and 24 h were 2.45-fold (*p* = 0.006) and 2.25-fold (*p* = 0.002) higher than that detected in the untreated control, respectively. The percentages of apoptotic cells detected in HK1 treated with 50 μM of H_2_O_2_ for 4 and 8 h were 1.68-fold (*p* = 0.009) and 2.18-fold (*p* = 0.007) higher than that detected in the untreated control, respectively (Fig. 3b i). Figure 3a ii and b ii are the representative contour plot diagrams showing the apoptotic population of H_2_O_2_-treated NP69 and HK1 cells, respectively.

### IPCR detection of chromosome breaks within the *ABL* gene mediated by stress-induced apoptosis

NP69 cells at confluency of 30–40% were treated with 10, 50 and 100 μM of H_2_O_2_ for 16 and 24 h while HK1 cells at optimal density were treated with 1, 10 or 50 μM of H_2_O_2_ for 2, 4, 6 and 8 h. For each cell line, an untreated sample was included to serve as a cell control. Nested IPCR was employed to identify chromosome breaks mediated by stress-induced apoptosis. The IPCR bands representing the *ABL* cleaved fragments detected were isolated, purified and sequenced.

**Fig. 4:**
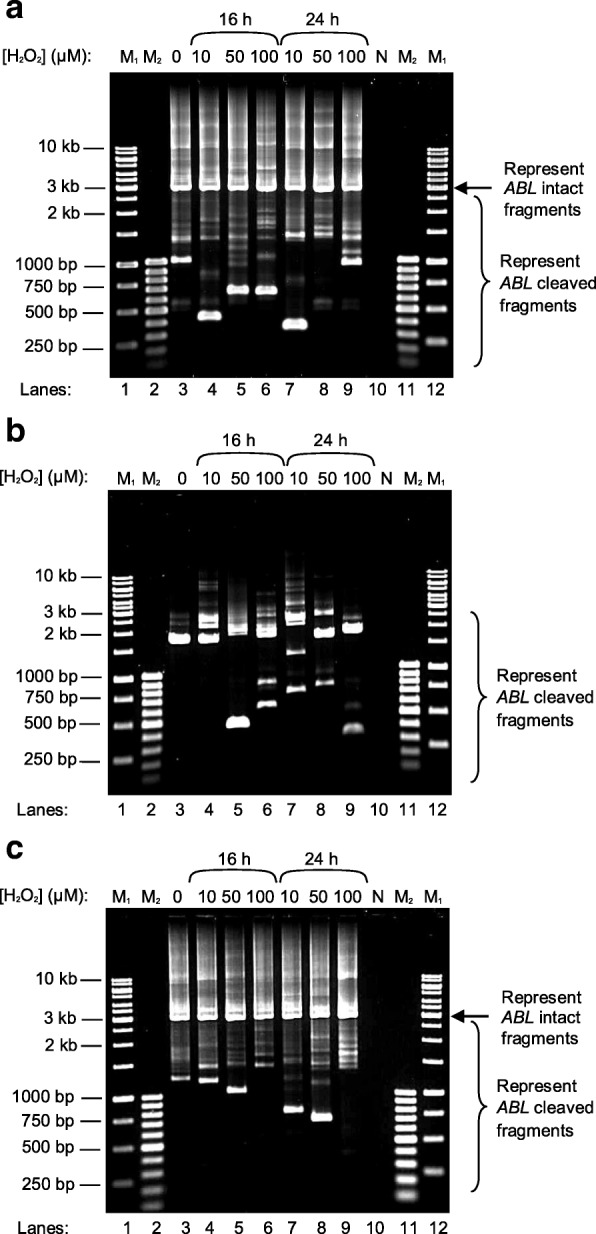
Nested IPCR detection of DNA breakages within the *ABL* gene in H_2_O_2_-treated NP69. NP69 cells at 30–40% confluency were either untreated (lane 3) or treated with 10 μM (lanes 4 and 7), 50 μM (lanes 5 and 8) or 100 μM (lanes 6 and 9) of H_2_O_2_ for 16 h (lanes 4–6) and 24 h (lanes 7–9). Genomic DNA was isolated and manipulated for nested IPCR. In the manipulation for nested IPCR, the DNA samples were subjected to digestion with *Age* I (**a**), double digestion with *Age* I and *Eco*R I (**b**) or double digestion with *Age* I and *Bsa*A I (**c**). The IPCR products were analysed on 1% agarose gel. Side arrows in panels **a** and **c** indicate the position of the 3 kb IPCR bands resulting from the amplification of the intact *ABL* gene. Side brackets in panels **a**, **b** and **c** indicate the possible IPCR bands from the *ABL* cleaved fragments. Negative control for PCR was included (lane 10). This IPCR result is representative of 2 repeats with similar results. M_1_: 1 kb DNA ladder. M_2_: 100 bp DNA ladder

**Fig. 5:**
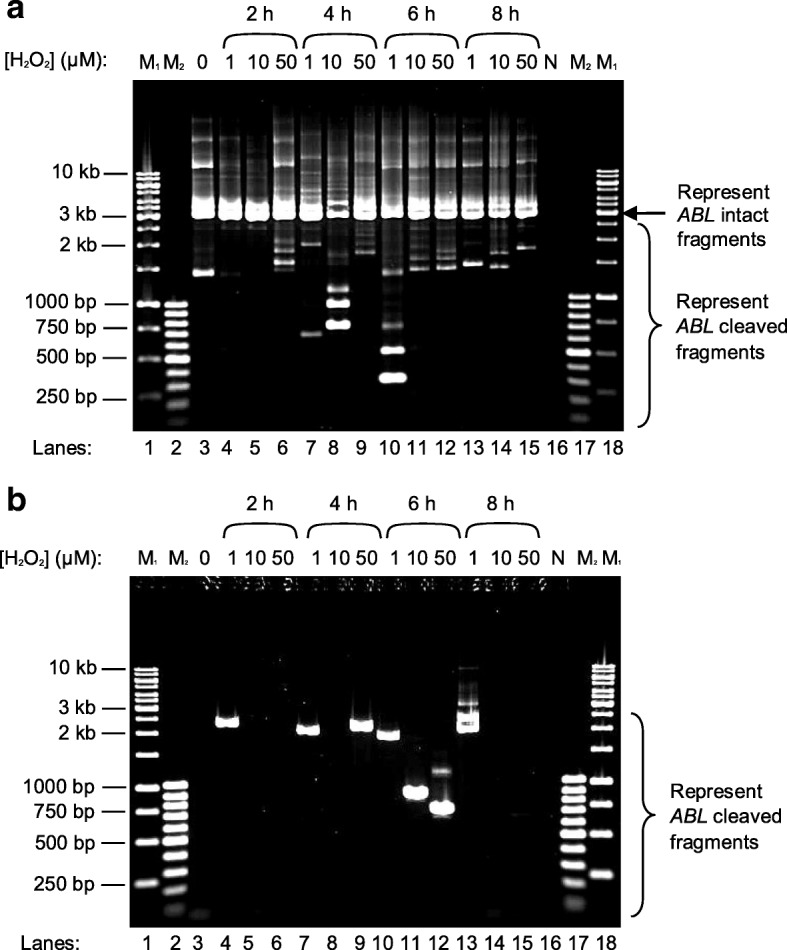
Nested IPCR detection of DNA breakages within the *ABL* gene in H_2_O_2_-treated HK1. HK1 cells were seeded in 60-mm culture dishes and were grown to optimal density (60–70% confluency). The cells were then either untreated (lane 3) or treated with 1 μM (lanes 4, 7, 10 and 13), 10 μM (lanes 5, 8, 11 and 14) or 50 μM (lanes 6, 9, 12 and 15) of H_2_O_2_ for 2 h (lanes 4–6), 4 h (lanes 7–9), 6 h (lanes 10–12) and 8 h (lanes 13–15). Genomic DNA was isolated and manipulated for nested IPCR. In the modification for nested IPCR, the DNA samples were either subjected to digestion with *Age* I (**a**) or double digestion with *Age* I and *Eco*R I (**b**). The IPCR products were analysed on 1% agarose gel. Side arrow in panel **a** indicates the position of the 3 kb IPCR bands resulting from the amplification of the intact *ABL* gene. Side brackets in both panels **a** and **b** indicate the possible IPCR bands from the *ABL* cleaved fragments. Negative control for PCR was included (lane 16). This IPCR result is representative of 2 repeats with similar results. M_1_: 1 kb DNA ladder. M_2_: 100 bp DNA ladder

Figures 4 and 5 show the IPCR results for H_2_O_2_-treated NP69 and HK1 cells, respectively. In the manipulation for nested IPCR, *Age* I (RE2 in Fig. 12) was used to linearise the cyclised DNA. If there is no breakage within the *ABL* gene, the IPCR product will be approximately 3 kb. On the contrary, if there is any breakage within the *ABL* gene, it should produce IPCR products which are smaller than 3 kb. As shown in Figures 4a and 5a, the *ABL* intact fragment of 3 kb is present in all of the samples. This amplification could serve as an internal control by proposing an optimal IPCR condition for the *ABL* gene. Besides, numerous IPCR bands of less than 3 kb were also obtained. However, these bands are less intense as compared with the intact fragment. This could be due to the competition between the intact fragments and the cleaved fragments for the amplification process. The intact fragments are usually more abundant as compared with the cleaved fragments. Consequently, the amplification of the cleaved fragments would be less efficient in the presence of the intact fragments. Therefore, double digestion with *Age* I and *Bsa*A I or *Age* I and *Eco*R I (RE3 in Fig. 12) was used to eliminate competition from the intact fragments for the nested IPCR reaction. These two different digestions gave rise to the detection of chromosome breaks within different regions. With double digestion of *Age* I and *Eco*R I, numerous distinct IPCR bands of less than 3 kb which represent the cleaved *ABL* fragment were detected in H_2_O_2_-treated NP69 (Fig. 4b, lanes 4–9) and H_2_O_2_-treated HK1 (Fig. 5b, lanes 4, 7, 9, 10, 11, 12 and 13) cells. A few cleavage bands were detected in the untreated NP69 cells (Fig. 4b, lane 1) which might be due to endogenous DNA breaks in the minority of untreated cells which were unhealthy. However, in general, there were more cleaved fragments detected in the treated samples compared with the untreated sample.

As shown in Fig. 4c, the intact fragment of 3 kb was still detected upon double digestion with *Age* I and *Bsa*A I. Most probably, this was due to incomplete digestion by *Age* I and *Bsa*A I. Regardless of the incomplete digestion, several cleavage bands of different sizes were detected in NP69 cells treated with various concentrations of H_2_O_2_ for different time points (Fig. 4c, lanes 4–9).

DNA breakages were detected in cell samples treated with various concentrations of H_2_O_2_ at various time points. Based on the microscopic analysis and flow cytometric analyses, the optimal concentration and time point were determined. These optimal concentration and time point were used to repeat the experiments in NP69 and HK1 cells. For NP69 cells, we selected concentration of 100 μM with exposure time of 16 and 24 h. The microscopic analysis on H_2_O_2_-treated NP69 cells showed that cytoplasmic shrinkage was only observed in cells treated with 100 μM for 16 and 24 h (Additional file 2). In addition, we performed flow cytometric analyses of PS externalisation and MMP loss on NP69 cells treated with 100 μM for 16 and 24 h. In these two flow cytometric assays, both samples showed significantly higher percentage of apoptosis as compared with that of the untreated control (Figs. 2a i and 3a i).

For HK1 cells, we chose concentration of 50 μM with exposure time of 8 h. Cell blebbing, which is one of the major morphological changes, was relatively more prominent in HK1 cells treated with 50 μM of H_2_O_2_ for 8 h compared with those treated with lower concentration (1 and 10 μM) and shorter exposure time (2, 4 and 6 h) (Additional file 3). Moreover, the flow cytometric analysis of PS externalisation on sample treated with 50 μM of H_2_O_2_ for 8 h showed a more promising result compared with the sample treated with 50 μM of H_2_O_2_ for 4 h (Fig. 2b i).

H_2_O_2_ treatment was thus repeated in NP69 and HK1 cells with the selected concentration and time point. NP69 cells at confluency of 30–40% were either untreated or treated with 100 μM of H_2_O_2_ for 16 h while HK1 cells at confluency of 60–70% were either untreated or treated with 50 μM of H_2_O_2_ for 8 h. The cells were then harvested for gDNA extraction and nested IPCR. In the manipulation for nested IPCR, all the samples were subjected to double digestion with *Age* I and *Eco*R I (RE3 in Fig. 12).

**Fig. 6:**
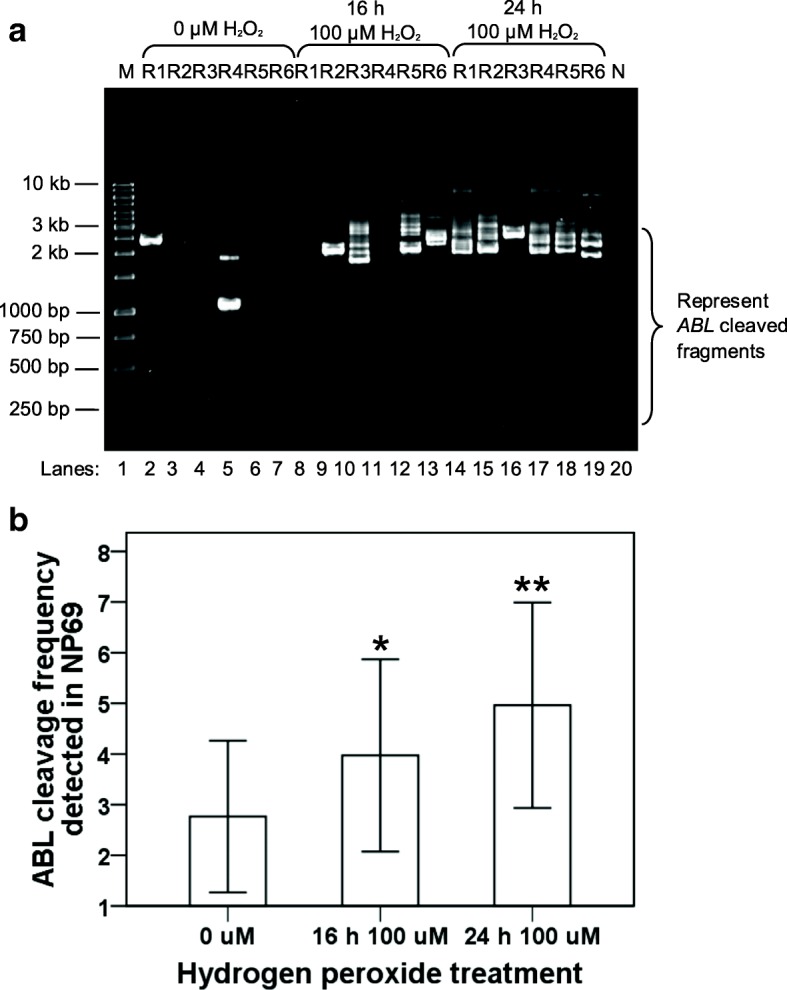
IPCR analysis of H_2_O_2_-induced chromosome breaks within the *ABL* gene in NP69 cells. **a** IPCR result obtained from H_2_O_2_-treated NP69 cells. NP69 cells were either untreated (lanes 2–7) or treated with 100 μM of H_2_O_2_ for 16 h (lanes 8–13) and 24 h (lanes 14–19). Genomic DNA was isolated and manipulated for nested IPCR. Double digestion with *Age* I and *Eco*R I was employed to eliminate competition of the intact fragments in the amplification process. Each cell sample consisted of six replicates (R1–6) in the nested IPCR. The IPCR products were analysed on 1.0% agarose gel. Side bracket indicates the possible IPCR bands derived from the *ABL* cleaved chromosome. Negative control for PCR was included (Lane 20). M: 100 bp DNA ladder. **b** The average number of DNA cleavage detected within the *ABL* gene. The data was expressed as means and SD of three independent experiments. Each experiment consisted of 1–3 sets of IPCR. Each set of IPCR was performed in 4–7 IPCR replicates for each cell sample. *p < 0.01, **p < 0.001 (Student’s *t* test)

Fig. 6a shows that numerous IPCR bands of less than 3 kb which represent the cleaved *ABL* gene detected in NP69 cells treated with H_2_O_2_ for 16 h (lanes 9, 10, 12 and 13) and 24 h (lanes 14–19). Three cleavage bands were identified in the untreated control (lanes 2 and 5). This might be due to spontaneous cell death of untreated cells as detected in our flow cytometric analyses. As shown in the bar chart in Fig. 6b, the cleavage frequencies of the *ABL* gene detected in NP69 cells treated with H_2_O_2_ for 16 and 24 h are 1.4-fold (*p* = 0.004966) and 1.8-fold (*p* = 0.000009) higher than that of the untreated control, respectively.

**Fig. 7:**
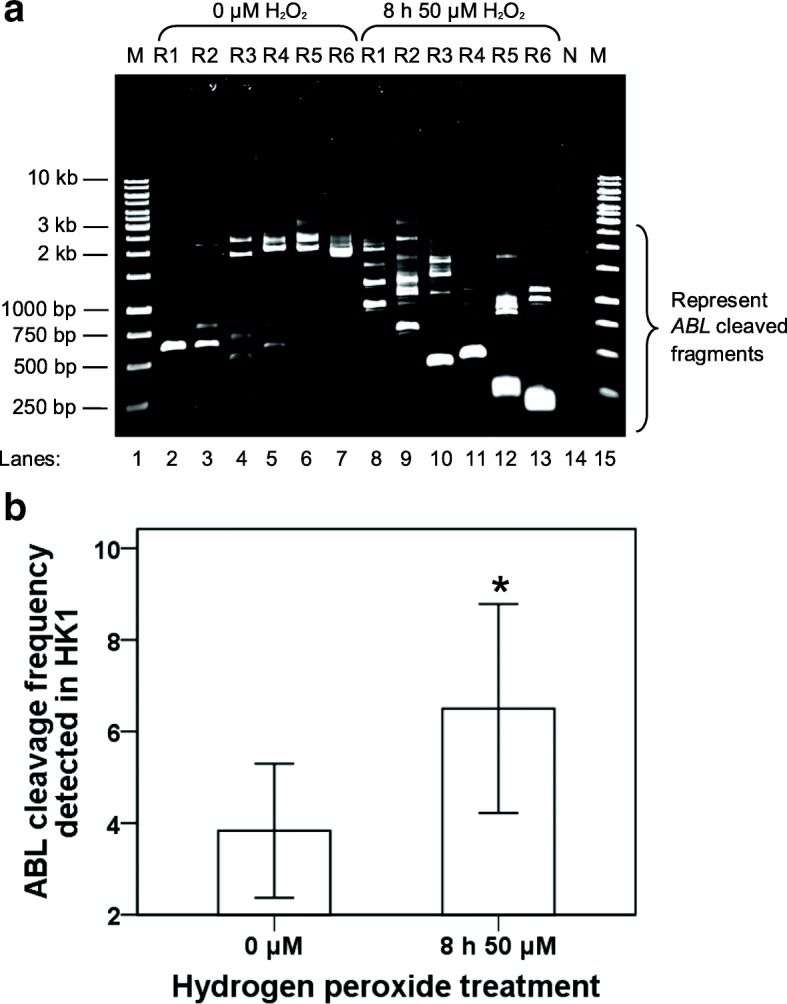
IPCR analysis of H_2_O_2_-induced chromosome breaks within the *ABL* gene in HK1 cells. **a** IPCR result obtained from H_2_O_2_-treated HK1 cells. HK1 cells were either untreated (lanes 2–7) or treated with 50 μM of H_2_O_2_ for 8 h (lanes 8–13). Genomic DNA was isolated and manipulated for nested IPCR. In the manipulation for nested IPCR, samples were subjected to double digestion with *Age* I and *Eco*R I to eliminate the competition of the intact fragments for amplification process. Each cell sample consisted of six replicates in nested IPCR. The IPCR products were analysed on 1.0% agarose gel. Side bracket indicates the possible IPCR bands derived from the *ABL* cleaved chromosome. Negative control for PCR was included (lane 14). M: 100 bp DNA ladder. **b** The average number of DNA cleavage detected within the *ABL* gene. The data was expressed as means and SD of three independent experiments. Each experiment consisted of 1–3 sets of IPCR. Each set of IPCR was performed in 6 IPCR replicates for each cell sample. *Pp< 0.001 (Student’s *t* test)

Similar findings were obtained from the H_2_O_2_ treatment of HK1 cells. Figure 7a is a representative gel picture showing the IPCR result obtained from this experiment. The untreated HK1 cells show a few cleavage bands (lanes 2–7) which most likely due to spontaneous cell death. In contrast, there were numerous cleavage bands identified in the H_2_O_2_-treated sample (lanes 8–13). The chart in Fig. 7b shows that the cleavage frequency of the *ABL* gene detected in H_2_O_2_-treated HK1 cells is 1.7-fold higher than that of untreated HK1 cells (*p* = 0.000197).

### Sequencing results

**Fig. 8:**
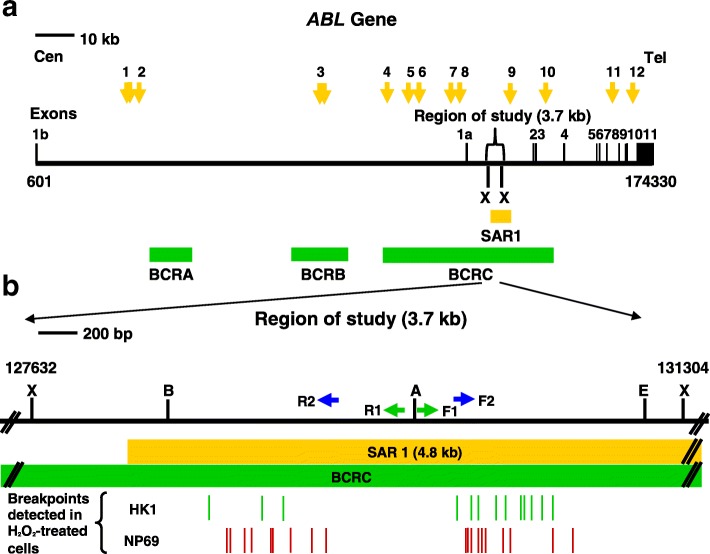
A map representing the positions of H_2_O_2_-induced chromosome breaks within the *ABL* gene. **a** The *ABL* genomic map from nucleotide positions 601-174330 is illustrated above [Ensembl:ENSG00000097007]. The locations of exons 1–11 are shown. The green boxes indicate the three previously identified patient breakpoints cluster regions which are designated as BCRA, BCRB and BCRC. The yellow box shows the previously biochemically extracted MAR/SAR which is indicated as SAR1 [77]. The yellow arrows represent the potential MAR/SARs predicted by MRS in this study. **b** The region of study (3.7 kb). *Xba* I (X), *Bsa*A I (B), *Age* I (A) and *Eco*R I (E) restriction sites are shown. The green and blue arrows represent the primers used in the first and second rounds of nested IPCR, respectively. The breakpoints identified in H_2_O_2_-treated HK1 and NP69 cells are indicated by the green and red vertical lines, respectively. All chromosome breaks were mapped within SAR1

In order to confirm that these fragments were derived from the cleaved *ABL* gene, some of the cleavage IPCR bands were extracted and sequenced. The sequencing results show that they were all derived from the cleaved *ABL* gene. Table 2 shows the breakpoints identified within the *ABL* gene in the H_2_O_2_-treated cells. A map illustrating the positions of chromosome breaks in HK1 and NP69 cells relative to the MAR/SAR sequences within the *ABL* gene is shown in Fig. 8.

**Table 2:** The chromosome breaks identified within the *ABL* gene in cells treated with H_2_O_2_

Cell line treated with H_2_O_2_	Breakpoint
NP69	129,265
	129,287
	129,372
	129,408
	129,520
	129,534
	129,628
	129,823
	130,633
	130,634
	130,638
	130,687
	130,699
	130,719
	130,822
	130,864
	131,108
	131,232
HK1	129,152
	129,461
	129,739
	130,653
	130,696
	130,791
	130,854
	130,921
	131,042

The nucleotide positions of the chromosome breaks identified within the *ABL* gene were mapped according to the *ABL* sequence retrieved from Ensembl database [Ensembl:ENSG00000097007]

**Fig. 9:**
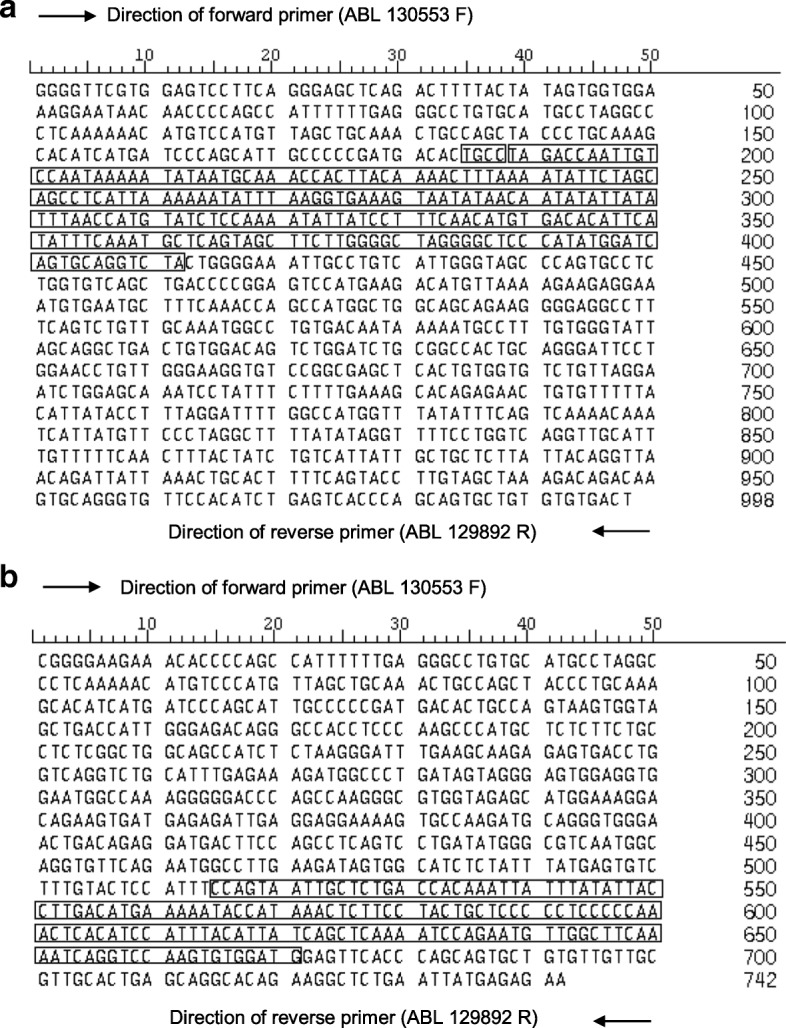
Shift translocations detected in H_2_O_2_-treated NP69 cells. **a** Treatment of NP69 with 100 μM of H_2_O_2_ for 16 h resulted in shift translocation. The DNA sequences 1–184 and 413–998 (without the box) represent the sequence derived from the *ABL* gene. The DNA sequence 185–412 (within the box) represents the sequence derived from the *LHFPL3* gene which locates at chromosome 7. Region of microhomology (185–188, TGCC) was found at the breakpoint junctions. The translocated fragment (228 bp) of *LHFPL3* gene is corresponding to coordinates 108,006–108,234 [Ensembl:ENSG00000187416]. **b** Treatment of NP69 with 10 μM of H_2_O_2_ for 24 h resulted in shift translocation. The DNA sequences 1–524 and 672–742 (without the box) represent the sequence derived from the *ABL* gene. The DNA sequence 525–671 (within the box) represents the sequence of the fragment translocated to the *ABL* gene. This translocated fragment (147 bp) is derived from chromosome 5. The disabled homologue 2 (*DAB*) gene is 1,263,556 bp at the 5′ end of this translocated fragment while a gene encoding for a hypothetical protein is 22,122 bp at the 3′ end

Intriguingly, we detected two shift translocations in H_2_O_2_-treated NP69 cells. Translocation is rarely observed in NPC, compared with deletion and addition. The first shift translocation was identified in NP69 cells exposed to 100 μM of H_2_O_2_ for 16 h. As shown in Fig. 9a, the translocated segment was derived from the human lipoma *HMGIC* fusion partner-like 3 (*LHFPL3*) gene which is located on chromosome 7. The *LHFPL3* gene consisting of three exons is 578,576 bp in length. The description of exons and introns in the *LHFPL3* gene is shown in Additional file 4. The translocated segment (228 bp) of the *LHFPL3* gene is corresponding to coordinates 108006–108234 [Ensembl:ENSG00000187416]. The breakpoints (108,006 and 108,234) of the *LHFPL3* gene were mapped within its first intron. Moreover, region of microhomology (TGCC) was found at the breakpoint junctions. The second shift translocation was identified in NP69 cells exposed to 10 μM of H_2_O_2_ for 24 h. The segment translocated to the *ABL* gene is derived from chromosome 5. The disabled homologue 2 (*DAB*) gene is 1,263,556 bp at the 5′ end of this segment while a gene encoding for hypothetical protein is 22,122 bp at the 3′ end (Fig. 9b).

## Discussion

Oxidative stress increases genomic instability [86] which in turn contributes to carcinogenesis [87, 88]. Excessive production of ROS has been associated with mutation and alteration of gene expression [49]. Most of the aetiological factors of NPC were known to generate ROS. These aetiological factors include exposures to nitrosamines, cigarette smoke, formaldehyde and wood dust. EBV infection as well as chronic inflammation of sinonasal tract [39, 41–43].

In addition, formaldehyde and acrolein, a component of cigarette smoke, are reactive aldehydes which may impair the function of DNA, RNA and proteins through adduct formation. It has been suggested that the combined interactions of environmental aldehydes and endogenous aldehydes, which are produced during oxidative stress, may exacerbate the cellular oxidative damage [47].

Although the consistent chromosomal aberrations, such as deletion and addition, have long been identified in NPC, the underlying molecular mechanism requires further investigation. Apoptosis was suggested to participate in the chromosomal translocation process of leukaemia [70]. Given that there is a strong association between the aetiological factors of NPC and oxidative stress, we intended to investigate the role of oxidative stress-induced apoptosis in mediating the chromosome rearrangements of NPC.

We demonstrated that hydrogen peroxide (H_2_O_2_), a strong oxidising agent, was able to induce apoptosis in normal nasopharyngeal epithelial cells (NP69) and NPC cells (HK1). Both exposure of PS and disruption of MMP are key events of apoptosis [89, 90]. By using flow cytometric analyses of PS externalisation and MMP loss, we detected significantly higher percentages of apoptosis in H_2_O_2_-treated NP69 and HK1 cells as compared with the untreated controls. As compared with NPC cells (HK1), longer exposure time and higher dosage of H_2_O_2_ were needed to trigger apoptosis in normal nasopharyngeal epithelial cells (NP69). There may be several possibilities that lead to this variation. The intrachromosomal instability in cancer cells is usually higher than that in normal cells [91, 92]. Furthermore, cancer cells may have a defective DNA repair system which is unable to restore the genomic integrity [91]. These factors imply that cancer cells are more susceptible to DNA damage. When apoptosis is triggered by oxidative stress, DNA fragmentation occurs. Cells try to survive apoptosis through DNA repair. Therefore, as compared with normal cells, cancer cells which have a higher intrachromosomal instability or a defective DNA repair system are usually more vulnerable to apoptosis.

In our previous report, we identified chromosomal breakages within the *AF9* gene in H_2_O_2_-treated NP69 and HK1 cells. In addition, inhibiting caspase-3 by caspase-3 inhibitor has abolished the *AF9* gene cleavages mediated by H_2_O_2_-induced apoptosis. Given that caspase-3 is the main activator of CAD-mediated DNA fragmentation in apoptosis, our findings suggested that CAD might be the major player which mediated the chromosomal breakages in H_2_O_2_-induced apoptosis [80]. It has been observed that CAD binds to the nuclear matrix during apoptosis [79]. Due to the fact that MAR/SAR sequences are the sites where DNA interacts with the nuclear matrix [93], it is likely that CAD cleaves the DNA at MAR/SAR sequences when it associates with nuclear matrix. Intriguingly, our previous report demonstrated that oxidative stress-induced apoptosis caused chromosomal breakages within the *AF9* BCR which is bordered by two MAR/SARs [80].

The present study targeted the *ABL* gene which is located on chromosome 9q34. This gene was targeted because 9q33-34 is one of the common deletion regions in NPC [23]. The *ABL* gene is the most common fusion partner gene with the breakpoint cluster region (*BCR*) gene which is located on chromosome 22q11 [94]. The reciprocal translocation t(9;22)(q34;q11) in CML was the first consistent chromosome rearrangement found in malignancy. The *ABL*-*BCR* fusion gene was named as the Philadelphia chromosome [95]. This reciprocal translocation was found in approximately 92% of CML patients. Thus, the *ABL*-*BCR* fusion gene is recognised as the cytogenetic hallmark of patients suffering from this disease [94, 96]. The presence of Philadelphia chromosome was also reported in 20 to 55% of adults and 2 to 10% of children with acute lymphoblastic leukaemia (ALL) [97] and rarely (1 to 2%) in acute non-lymphoblastic leukaemia (ANLL) [98]. There are three BCRs found within the *ABL* gene. The first BCR (BCRA) and the second BCR (BCRB) are located in intron 1b, whereas the third BCR (BCRC) spans through parts of introns 1b to 3. BCRC is the largest BCR of the *ABL* gene [77, 99].

One biochemically defined MAR/SAR has been previously identified within the BCRC of the *ABL* gene. This MAR/SAR was designated as SAR1. SAR1 was found within intron 1a [72]. In the present study, we predicted MAR/SAR sites within the *ABL* gene by using MRS which was proposed to be strongly associated with MAR/SAR [84]. It has been found that the two sequence elements of the MRS exist at a position near the dyad axis of the nucleosome. The wrapping of the DNA around the histone protein complex causes the two sequence elements of the MRS to be physically close together even if they are non-adjacent on the linear DNA. The close proximity between the two sequence elements of the MRS on the positioned nucleosome allows them to generate a protein-binding site in MAR/SAR [84].

The variation in the distance between the two sequence elements suggested a relation of the MRS to nucleosome organisation. In the *Drosophila* histone cluster, there was a MAR/SAR identified between the histone H1 and H3 genes. This MAR/SAR was found to contain a few nucleosomes and two MRSs. It was observed that the position of the two MRSs on their respective nucleosomes is similar. The first MRS, where the two sequence elements are overlapping, is found on the dyad axis of a nucleosome. The second MRS, where the two sequence elements are 145 bp apart, is located near the entry and exit sites of a nucleosome. Although the two sequence elements of the MRS are spatially distant, they are brought close together when the DNA is turned around the histone core [85].

A nucleosome comprises a nucleosome core and a ‘linker’ DNA. The nucleosome core contains 145–147 bp of DNA wrapped around a core histone octamer. The histone octamer consists of two molecules each of the four core histones, namely, H2A, H2B, H3 and H4 [94]. It has been known that the length of ‘linker’ DNA ranges from 15 to 100 bp, depending on the cell types. The ‘linker’ DNA connects one nucleosome to the other (reviewed in [95]). The nucleosome repeat length (NRL) refers to the length of nucleosomal DNA (145–147 bp) plus the length of linker DNA (15–100 bp) [94, 95]. Using the micrococcal nuclease assay, the NRL has been reported to range from 160 to 240 bp [96, 97].

In the studies by van Drunen et al. (1999), the distance between the two sequence elements of the MRS has been suggested to be within 200 bp [85]. SAR prediction/SAR prediction presently performed in the *ABL* gene, there was only one MAR/SAR site (MAR/SAR 9 in Table 1) predicted in the experimentally isolated SAR1. The distance between the 8 bp sequence element and the 16 bp sequence element was found to be 248 bp. Given that the NRL may exceed 200 bp, for the mapping of MRS in the present study, the maximal distance between the 8 bp sequence element and the 16 bp sequence element was set at 250 bp. Besides, it is also possible that the two sequence elements, which are 248 bp apart, are located separately on two adjacent nucleosomes. In the positioned nucleosomes, interaction between two adjacent nucleosomes may happen. Thus, it seems possible that even if the two sequence elements are individually located on two adjacent nucleosomes, the wrapping of DNA around the histone protein complex may still cause them to be physically close together and enable them to generate a protein binding site.

We predicted 12 potential MAR/SAR sites within the *ABL* gene. One MAR/SAR site was predicted within the biochemically defined SAR1. Interestingly, 10 out of these 12 (> 80%) potential MAR/SAR sites are closely associated with the BCRs of the *ABL* gene (Fig. 1). MAR/SARs 1 and 2 were predicted next to BCRA. MAR/SAR 3 was found within BCRB. MAR/SARs 4 to 10 were identified within BCRC.

By using IPCR, we identified chromosome breaks in H_2_O_2_-treated NP69 and HK1 cells. The cleavage frequency of the *ABL* gene in H_2_O_2_-treated cells was significantly higher than that in the untreated control cells. This is true for both NP69 and HK1 cell lines. These results reaffirm our previous findings which demonstrated that oxidative stress-induced apoptosis resulted in chromosomal breakages in normal nasopharyngeal epithelial and NPC cells [80]. Taken together, our findings are consistent with other studies which discovered that H_2_O_2_ induced apoptotic DNA fragmentation. It has been demonstrated that H_2_O_2_ induced excision of chromosomal DNA loops mediated by topoisomerase II in U937 leukaemic cells [100]. The production of these HMW DNA fragments (50–100 kb loop-sized DNA fragments) is an initial event of apoptosis [65]. It has also shown that in caspase-3-expressing MCF-7 breast carcinoma cells, H_2_O_2_ activated DNA fragmentation with nucleosomal intervals [101]. The fragmentation of nuclear DNA into nucleosomal DNA ladders is another hallmark of apoptosis [102].

Our sequencing results have confirmed that the IPCR bands were derived from the cleaved *ABL* gene. All of the breakpoints were mapped within the biochemically defined SAR1 of the *ABL* gene. SAR1 is located in BCRC, the largest BCR of the *ABL* gene [77]. MAR/SAR is thought to be one of the common chromatin structures within BCRs. The BCRs of *AF9*, *MLL* and *AF4* genes have all been found to associate with MAR/SAR, suggesting a role for MAR/SAR in non-homologous recombination (NHR) [76–78]. MAR/SAR sequences were found to possess DNA unwinding properties [103, 104]. These properties allow them to facilitate the entry of protein factors that take part in chromosome condensation, apoptosis, transcription and replication [104, 105]. However, these unwinding properties also cause MAR/SAR sequences to be more prone to DNA breakage [103, 104].

In addition, two shift translocations were detected in H_2_O_2_-treated NP69 cells. One of the translocated segments was derived from the *LHFPL3* gene which locates at chromosome 7.

The other translocated segment was derived from chromosome 5. The disabled homologue 2 (*DAB*) gene is 1,263,556 bp at the 5′ end of this translocated segment while a gene encoding for hypothetical protein is 22,122 bp at the 3′ end.

The *LHFPL3* gene is one of the family members of LHFP-like genes. This gene family consists of six family members. All of the family members have been implicated in human diseases. Members of this family are transmembrane proteins which play important roles in extracellular matrix formation, differentiation and proliferation. Most of them have been associated with tumours [106]. The first member, *LHFP* on chromosome 13q12, was identified, for the first time, as a translocation partner of *HMGIC* gene on chromosome 12q15 in human lipoma with t(12;13)(q15;q12). Thus, it was annotated as lipoma *HMGIC* fusion partner (*LHFP*) gene [107]. The *LHFPL1* gene on chromosome Xq23 has been implicated in liver tumour [108]. The *LHFPL2* gene on chromosome 5q14.1 was found to be highly expressed in the novel subgroup of ALL [109, 110] and in patients who succumbed fatally to serous epithelial ovarian cancers (SEOC) [111]. The *LHFPL4* gene at 3p25.3 was identified as a novel methylation target specific for cervical cancer [112]. Mutation in the *LHFPL5* gene (on chromosome 6p21.31) which is also known as tetraspan membrane protein of hair cell stereocilia (*TMHS*) gene has been found to cause autosomal recessive nonsyndromic deafness [113].

The *LHFPL3* gene is located on chromosome 7q22.1. Deletions involving chromosome 7q22 are commonly observed in uterine leiomyoma (UL). Four distinct deletion intervals have been identified. One of the microdeletions contains the *LHFPL3* gene*.* The single deleted marker in the microdeletion was mapped within the first intron of the *LHFPL3* gene. These findings suggested that the *LHFPL*3 gene is a candidate tumour suppressor gene (TSG) for UL [106]. Deletion of 7q22 has also been associated with leukaemia. A commonly deleted segment of chromosome 7q22 has been identified in patients with a malignant myeloid disease. The *LHFPL3* gene is one of the candidate TSGs residing in this deletion interval [114]. More recently, the alteration of *LHFPL3* gene has been suggested to be a hallmark of primary glioblastoma [115].

**Fig. 10:**
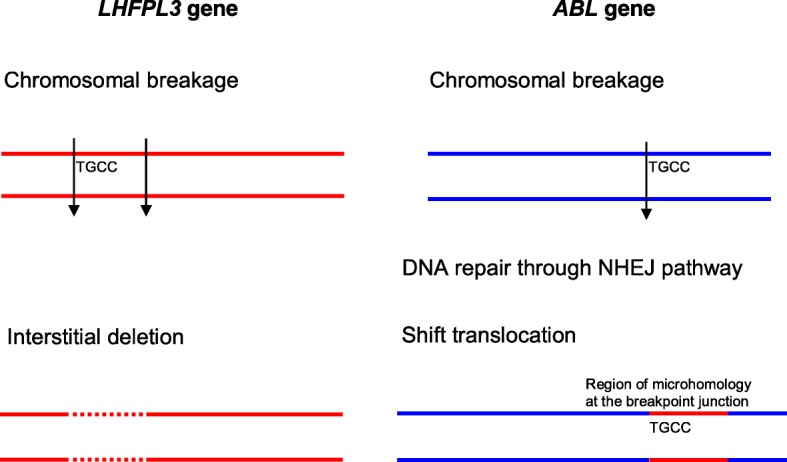
A potential model for the shift translocation of the *LHFPL3* gene. During oxidative stress-induced apoptosis, chromosomal breakages occur within both the *LHFPL3* (located at chromosome 7q22) and *ABL* (located at chromosome 9q34) genes. Following that, interstitial deletion occurs within the *LHFPL3* gene. When the cells try to survive apoptosis, DNA repair takes place. By utilising the region of microhomology, TGCC, that was found at the breakpoint junctions of both the *LHFPL3* and *ABL* genes, the two DNA ends were joined. Subsequently, cells that survive apoptosis may carry the *ABL* gene with the shift translocation of a segment of the *LHFPL3* gene

Intriguingly, region of microhomology (four nucleotides) was found at the breakpoint junctions. This observation suggested that the shift translocation of the *LHFPL3* gene might be mediated by NHEJ DNA repair pathway. Based on the analysis of our sequencing data, we illustrated the potential model for the shift translocation of the *LHFPL3* gene (Fig. 10). As proposed by Betti and colleagues (2001), the interaction of the NHEJ DNA repair pathway with apoptosis can act as a mechanism leading to translocation in leukaemia. They found that translocation junctions between the *MLL* gene and the partner DNA contain regions of microhomology consistent with the operation of NHEJ repair process [70]. In addition, it has been found that cells that survive apoptosis may contain rearranged chromosomes that contribute to leukaemogenesis [69]. Taken together, the findings of ours and of others support the notion that the interaction of the NHEJ DNA repair system with oxidative stress-induced apoptosis may be a possible mechanism leading to chromosome rearrangements in NPC.

**Fig. 11:**
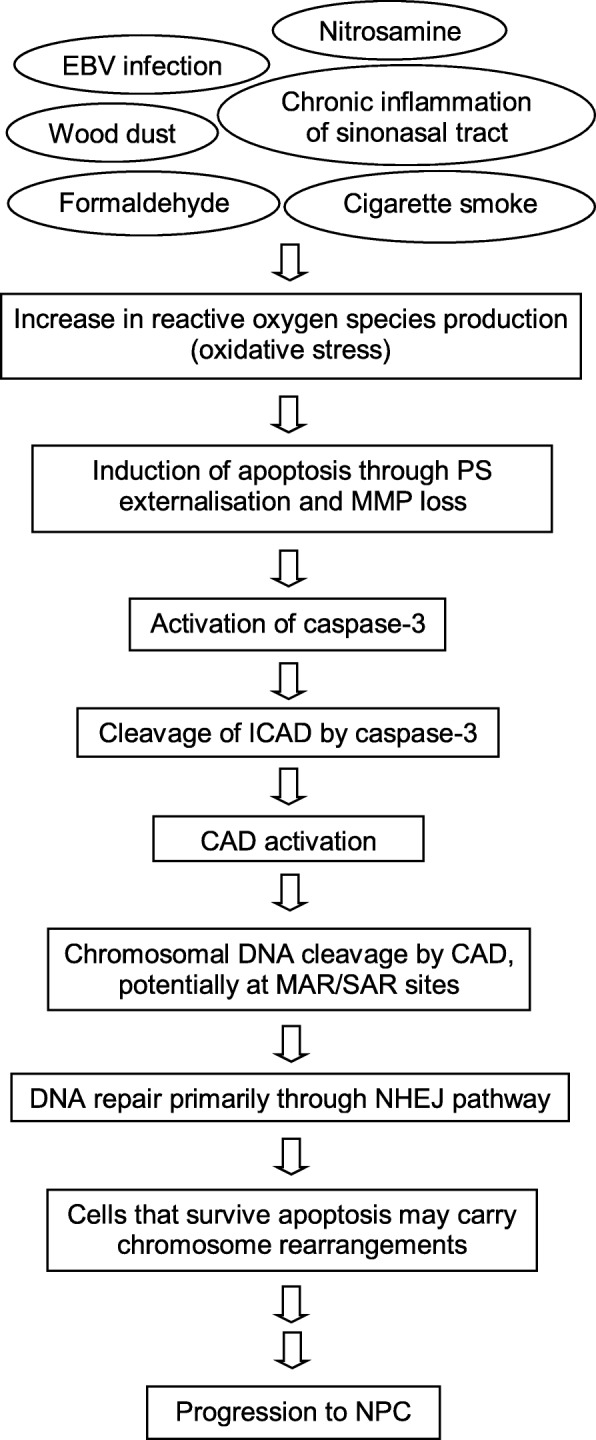
A revised model for oxidative stress-induced chromosome rearrangement in NPC

We previously proposed a potential model for oxidative stress-induced chromosome rearrangements in NPC involving the *AF9* gene [80]. Based on the findings of the *ABL* gene in the present study and additional findings from the literature, we proposed a revised model (Fig. 11). The revision of this model enables us to further elucidate the potential role of oxidative stress-induced apoptosis in mediating chromosome rearrangements in NPC. We propose that oxidative stress plays an essential role in NPC aetiological factors. These include exposure to nitrosamine, wood dust, formaldehyde and cigarette smoke. EBV infection as well as chronic inflammation of sinonasal tract. Oxidative stress-induced apoptosis is initiated by apoptotic signalling. This includes PS externalisation and MMP loss. The apoptotic signalling may in turn result in the activation of the main effector caspase, caspase-3. Caspase-3 cleaves ICAD that contains two caspase-3 cleavage sites. Subsequently, CAD is being released from its chaperone, ICAD. Chromosomal DNA is cleaved by the activated CAD, presumably at MAR/SAR sites. Double strand breaks are primarily repaired through NHEJ pathway which is prone to cause erroneous DNA repair. Cells that evade apoptosis may harbour chromosome rearrangements such as translocation, deletion, addition and inversion. Repeated exposure to these aetiological factors that provoke oxidative stress may therefore contribute to tumourigenesis of NPC.

In the present study, we only focused on the SAR region of the *ABL* gene. It is difficult to draw a solid conclusion on the role of MAR/SAR in defining the positions of the chromosome breakages. Therefore, for the future work, comparison in the cleavage frequency between the *ABL* SAR region and non-SAR region may be carried out. This may allow a further elucidation of the potential role of MAR/SAR in mediating the chromosome breakages and rearrangements in oxidative stress-induced apoptosis.

## Conclusions

Our findings demonstrated that oxidative stress-induced apoptosis may be a potential mechanism that leads to chromosome rearrangements in NPC. Our results also suggested that NHEJ system is potentially involved in DNA repair in cells undergoing oxidative stress-induced apoptosis. The interaction between NHEJ DNA repair system and oxidative stress-induced apoptosis may lead to chromosome rearrangements in surviving cells. A revised model for oxidative stress-induced apoptosis in mediating chromosome rearrangement in NPC is proposed.

## Methods

### Cell lines

NP69 normal nasopharyngeal epithelial cell line and HK1 NPC cell line were kindly provided by Prof. Tsao Sai Wah (The University of Hong Kong, Hong Kong, China) and Prof. Lo Kwok Wai (The Chinese University of Hong Kong, Hong Kong, China). NP69 is an immortalised nasopharyngeal epithelial cell line which was established by transfection with SV40 large T oncogene. It retains some characteristics of normal nasopharyngeal epithelial cells and is non-tumourigenic. This cell line may provide potential nasopharyngeal epithelial cell model for investigating mechanisms involved in NPC tumourigenesis [116]. HK1 was derived from a Chinese male patient with recurrent squamous NPC 17 ½ years after radiation therapy [117].

### Chemicals

Hydrogen peroxide (H_2_O_2_) was bought from MP Biomedicals, USA. Keratinocyte-SFM medium, RPMI 1640 medium, penicillin, streptomycin, fetal bovine serum and l-glutamine were purchased from GIBCO, Invitrogen, USA. Annexin V-Fluorescein isothiocyanate (FITC) Apoptosis Detection Kit I (BD Pharmingen™) and Flow Cytometry Mitochondrial Membrane Potential Detection Kit were bought from BD™ MitoScreen, Becton–Dickinson Biosciences, USA. Camptothecin (CPT) was purchased from Santa Cruz Biotechnology, CA, USA. Ammonium acetate was bought from Merck, Germany. Chloroform was bought from R&M Chemicals, UK. Phenol and Sodium dodecyl sulfate (SDS) were procured from Amresco, USA. Isoamyl alchohol was purchased from Fluka, Switzerland. Phusion High-Fidelity DNA Polymerase was procured from Finnzymes, Finland. PCR primers were from First Base Laboratories. QIAquick Gel Extraction Kit and QIAquick Nucleotide Removal Kit were bought from QIAGEN, Germany. DNA Polymerase I Large (Klenow) Fragment, restriction enzymes and T4 DNA Ligase were obtained from New England Biolabs (NEB), USA. dNTP mix was purchased from Promega, USA.

### Cell cultures

NP69 cells were grown in Keratinocyte-SFM medium supplemented with 100 μg/ml streptomycin, 100 U/ml penicillin, 40–50 μg/ml Bovine Pituitary Extract (BPE), 4–5 ng/ml recombinant Epidermal Growth Factor (rEGF) and 2% (*v*/*v*) heat-inactivated fetal bovine serum. HK1 cells were cultured in RPMI 1640 medium supplemented with 100 μg/ml streptomycin, 100 U/ml penicillin, 2 mM l-glutamine and 10% (*v*/*v*) heat-inactivated fetal bovine serum. Cells were cultured at 37 °C with 5% CO_2_.

### In silico prediction of MAR/SAR

The whole sequence of the *ABL* gene was retrieved from Ensembl (http://www.ensembl.org/index.html) database [Ensembl:ENSG00000097007]. The location of the experimentally defined MAR/SAR was determined from the previous report [77]. By using DNASTAR software (Lasergene, USA), we predicted the possible MAR/SAR sites within the *ABL* gene. The prediction of MAR/SAR site was performed by searching MRS which comprises two nucleotide motifs. The first nucleotide motif is an 8 bp degenerate sequence, AATAAYAA, where Y = C or T. The second nucleotide motif is a 16 bp degenerate sequence, AWWRTAANNWWGNNNC, where N = A, C, G or T; R = A or G; W = A or T. One mismatch is allowed in the 16 bp degenerate sequence. The 8 bp degenerate sequence has to be exactly matched. The two sequence elements of the MRS should be found within 200 bp apart. The two sequence elements can be present on either Watson or Crick strand and in either order. The two sequence elements may also be overlapping. When there are more than one motif of either 8 or 16 bp found within a distance of 200 bp, they are considered as a single MRS. In addition, when there is more than one MRS identified within close proximity, they are regarded as a single potential MAR/SAR site [85].

### Apoptosis detection

#### Phosphatidylserine (PS) externalisation

NP69 cells (1.5 × 10^5^) were plated in 150-mm culture dishes containing 15 ml of complete media. When NP69 cells reached confluency of 30–40% on the third day, NP69 cells were either left untreated or treated with 100 μM of H_2_O_2_ for 16 and 24 h. HK1 cells (5.5 × 10^5^) were seeded in 150-mm culture dishes containing 15 ml of complete media. When HK1 cells reached confluency of 60–70% on the fourth day, HK1 cells were incubated with 50 μM of H_2_O_2_ for 4 and 8 h. NP69 and HK1 cells treated with camptothecin (CPT) were included as positive controls. After incubation, the cells were collected by using StemPro ACCUTASE Cell Dissociation Reagent. The percentage of apoptotic cells was determined by using Annexin V-FITC Apoptosis Detection Kit I as previously described [80].

#### Mitochondrial membrane potential (MMP) loss

NP69 and HK1 cells were treated and collected as described above. The percentage of MMP loss in the harvested cells was determined by using Flow Cytometry Mitochondrial Membrane Potential Detection Kit as previously described [80].

### IPCR detection of chromosome breaks within the *ABL* gene

#### Induction of apoptosis in normal nasopharyngeal epithelial and NPC cells with H_2_O_2_

NP69 (2 × 10^4^) and HK1 (8 × 10^4^) cells were seeded in 60-mm culture plates containing 4 ml of complete media. When NP69 cells reached confluency of 30–40% on the third day, NP69 cells were either left untreated or treated with 10, 50 or 100 μM for 16 and 24 h. When HK1 cells reached confluency of 60–70% on the fourth day, HK1 cells were either left untreated or treated with 1, 10 or 50 μM of H_2_O_2_ for 2, 4, 6 and 8 h.

#### Genomic DNA extraction

At the end of the indicated exposure times, the used medium was discarded. The cells were washed once with cold 1× phosphate-buffered saline (PBS). Genomic DNA extraction was performed as previously described [80].

#### Manipulation of the extracted gDNA for nested IPCR

**Fig. 12:**
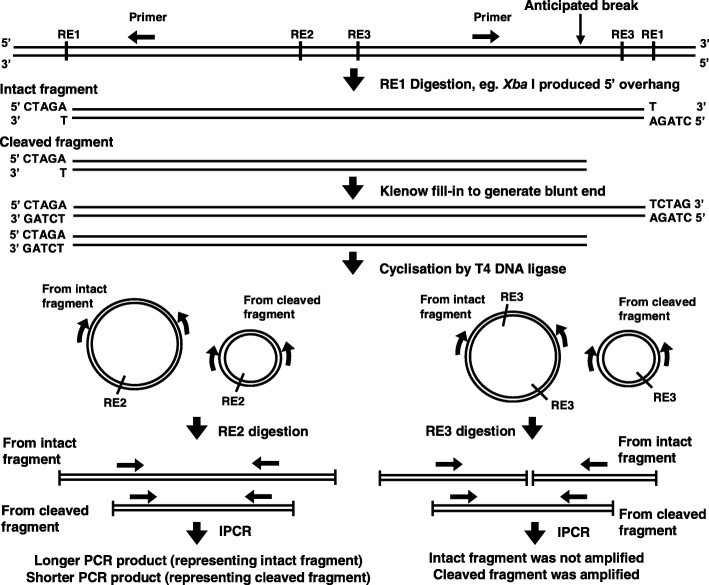
A flowchart showing the manipulation steps in the preparation of genomic DNA for IPCR. The genomic DNA was subjected to RE digestions, Klenow fill-in and ligation prior to IPCR as reported before [80]

The extracted gDNA was manipulated as described previously [80] with minor modifications. Figure 12 shows the manipulation steps. Digestion of the gDNA was performed at 37 °C for 16 h with 100 U of *Xba* I (RE1 in Fig. 12). The staggered four base pairs (CTAG) 5′ overhang was produced by *Xba* I digestion. The blunt ends were generated by the apoptotic nuclease such as CAD [118]. After *Xba* I digestion, both ends of the intact targeted DNA fragment were *Xba* I sites with staggered overhangs. As for the cleaved targeted DNA fragment, one end was the blunt end produced by the apoptotic nuclease, and the other end was the staggered overhang generated by *Xba* I. To produce blunt-ended fragments, Klenow fill-in was performed with two μg of DNA template, two units of DNA Polymerase I Large (Klenow) Fragment and 33 μM of dNTP mix at 25 °C for 15 min. Cyclisation was then performed with 2000 U of T4 DNA ligase at 16 °C for 16 h. Ethanol precipitation was carried out with 3 M sodium acetate (NaAc) (one volume), pH 5.2 and ice cold absolute ethanol (2.5 volumes). Seventy percent ethanol was used to wash the DNA pellet. The DNA pellet was then air-dried and dissolved in TE, pH 8.0. The DNA sample was divided into three. The DNA samples of tubes 1, 2 and 3 were subjected to digestion with 10 U of *Age* I (RE2 in Fig. 12), double digestion with 10 U of each *Age* I and *Bsa*A I (RE3 in Fig. 12), and double digestion with 10 U of each *Age* I and *Eco*R I (RE3 in Fig. 12), respectively. These RE digestions were performed at 37 °C for 16 h. Digestion with *Age* I was used to linearise the cyclised DNA. Double digestion with *Age* I and *Bsa*A I or *Age* I and *Eco*R I was used to eliminate competition from the intact fragments during IPCR. The double digestion with *Age* I and *Bsa*A I enabled the detection of DNA cleavages occurred within the amplified region towards the 3′ end. The double digestion with *Age* I and *Eco*R I enabled the detection of DNA cleavages occurred within the amplified region towards the 5′ end. According to the manufacturer’s protocol, QIAquick Nucleotide Removal Kit (QIAGEN) was used to purify the digested DNA.

#### Nested IPCR

The optical density (O.D.) of the purified DNA sample was measured by using an ultraviolet-visible micro-volume spectrophotometer (ND-1000, NanoDrop, USA). Nested IPCR was performed with 1× of HF buffer (containing 1.5 mM of MgCl_2_), 0.5 μM of each reverse primer and forward primer, 200 μM of dNTP mix, 0.4 U of Phusion High-Fidelity DNA Polymerase and 200 ng of DNA template. To serve as a negative control, sterile ultrapure water was used to replace the DNA template. Cycle condition used in the first round was: 30 s of 98 °C for 1 cycle (initial denaturation), followed by 30 cycles of 98 °C for 10 s (denaturation), 64 °C for 30 s (annealing), 72 °C for 55 s (extension), followed by 1 cycle of 72 °C for 10 min (final extension). Similar cycle condition was used in the second round of IPCR, except that the extension time was 50 s. Two microlitres of 5-fold diluted IPCR product of the first round was used as DNA template. The primers used in the first round of IPCR were 5’-GGTACCTGGTGTCTGTCTCTATC-3′ (reverse) and 5′-AGAAGGTTTATGGGAGATGG-3′ (forward), whereas the primers used in the second round were 5′-TCTCTCATATCTCAGAGCCTTC-3′ (reverse) and 5′-CTTCAGGAGCTCAGACTTTTAC-3′ (forward). The IPCR assays were done by using a Veriti 96 Well Thermal Cycler (Applied Biosystems, USA).

### Agarose gel electrophoresis and DNA sequencing

The PCR products were analysed on 1% agarose gel. The agarose gel electrophoresis was performed at 90 V for 1 h and 30 min. The agarose gel was briefly stained with ethidium bromide (0.5 μg/ml) and destained with distilled water. This was followed by visualisation of the gel on an ultraviolet (UV) transilluminator (Vilber Lourmat). The gel image was captured and analysed using a gel documentation (gel doc) and image analysis system (Syngene). The IPCR bands representing cleaved DNA fragments of the *ABL* gene were purified by using QIAquick Gel Extraction Kit (QIAGEN) according to the manufacturer’s protocol and sequenced. By blasting the human genome database (Genomic BLAST, https://blast.ncbi.nlm.nih.gov/Blast.cgi), the sequencing data obtained was annotated. To identify the breakpoints of the cleaved fragments, the sequencing data was analysed and aligned with the published *ABL* gene sequence [Ensembl:ENSG00000097007] by using Seqman DNASTAR software (Lasergene, USA). The positions of DNA breaks identified were compared with the location of the MAR/SAR sequence isolated experimentally in the previous study [77] and the MRS identified in the present study. A genomic map was constructed to depict the positions of the detected DNA breaks relative to the location of the MAR/SAR.

### Quantification of gene cleavage frequency

In each set of IPCR, four to seven IPCR replicates were prepared per cell sample. Each experiment consisted of one to three sets of IPCR. The number of IPCR bands representing the *ABL* cleaved fragments was counted. Gene cleavage frequency expresses the average number of *ABL* cleaved fragments detected in three independent experiments.

### Statistical analysis

Experiments were repeated three to five times. The significance of differences in the gene cleavage frequency detected by nested IPCR was evaluated by Student’s *t* test. Data for IPCR are expressed as mean and standard deviation (SD). Differences were considered statistically significant at *p* value < 0.05. All statistical tests are two sided.

## Additional files

Additional file 1: Description of exons and introns in the *ABL* gene. (PDF 65 kb)

Additional file 2: Microscopic images of NP69 cells after treatment with H_2_O_2_. (PDF 107 kb)

Additional file 3: Microscopic images of HK1 cells after treatment with H_2_O_2_. (PDF 150 kb)

Additional file 4: Description of exons and introns in the *LHFPL3* gene. (PDF 56 kb)

## Abbreviations

ALL: Acute lymphoblastic leukaemia
BCR: Breakpoint cluster region
CAD: Caspase-activated deoxyribonuclease
CML: Chronic myelogenous leukaemia
*DAB*: Disabled homologue 2
DSB: DNA double-strand breaks
EBV: Epstein-Barr virus
H_2_O_2_: Hydrogen peroxide
HMW: High-molecular-weight
HR: Homologous recombination
ICAD: Inhibitor of caspase-activated deoxyribonuclease
IPCR: Inverse polymerase chain reaction
*LHFPL3*: Human lipoma HMGIC fusion partner-like 3
MAR/SAR: Matrix association region/scaffold attachment region
*MLL*: Mixed lineage leukaemia
MMP: Mitochondrial membrane potential
NHEJ: Non-homologous end joining
NPC: Nasopharyngeal carcinoma
PS: Phosphatidylserine
ROS: Reactive oxygen species
UL: Uterine leiomyoma
