# Supplementary material for: Oxidative stress-induced chromosome breaks within the ABL gene: a model for chromosome rearrangement in nasopharyngeal carcinoma
Source: Hum Genomics. 2018 Jun 18;12:29. doi: 10.1186/s40246-018-0160-8 (PMC6006577; doi:10.1186/s40246-018-0160-8)
Supplement: Supplementary file 1 — Description of exons and introns in the ABL gene. (PDF 65 kb) [file 40246_2018_160_MOESM1_ESM.pdf]

Additional file 1

Description of exons and introns in the *ABL* gene

Exon/intron	*Nucleotide position*	*Length (bp)*
5' upstream sequence	1-600	600
Exon 1b	601-1171	571
1st Intron 1	1172-121720	120549
Exon 1a	121721-122180	460
2nd Intron 1	122181-140718	18538
Exon 2	140719-140892	174
Intron 2	140893-141455	563
Exon 3	141456-141751	296
Intron 3	141752-149417	7666
Exon 4	149418-149690	273
Intron 4	149691-158783	9093
Exon 5	158784-158868	85
Intron 5	158869-159514	646
Exon 6	159515-159692	178
Intron 6	159693-161522	1830
Exon 7	161523-161707	185
Intron 7	161708-165069	3362
Exon 8	165070-165222	153
Intron 8	165223-166722	1500
Exon 9	166723-166812	90
Intron 9	166813-167100	288
Exon 10	167101-167319	219
Intron 10	167320-170623	3304
Exon 11	170624-174330	3707
3'downstream sequence	174331-174930	600

The *ABL* gene located at 9q34 is 173795 bp in length [Ensembl:ENSG00000097007].
