# Supplementary material for: Oxidative stress-induced chromosome breaks within the ABL gene: a model for chromosome rearrangement in nasopharyngeal carcinoma
Source: Hum Genomics. 2018 Jun 18;12:29. doi: 10.1186/s40246-018-0160-8 (PMC6006577; doi:10.1186/s40246-018-0160-8)
Supplement: Supplementary file 2 — Microscopic images of NP69 cells after treatment with H2O2. (PDF 107 kb) [file 40246_2018_160_MOESM2_ESM.pdf]

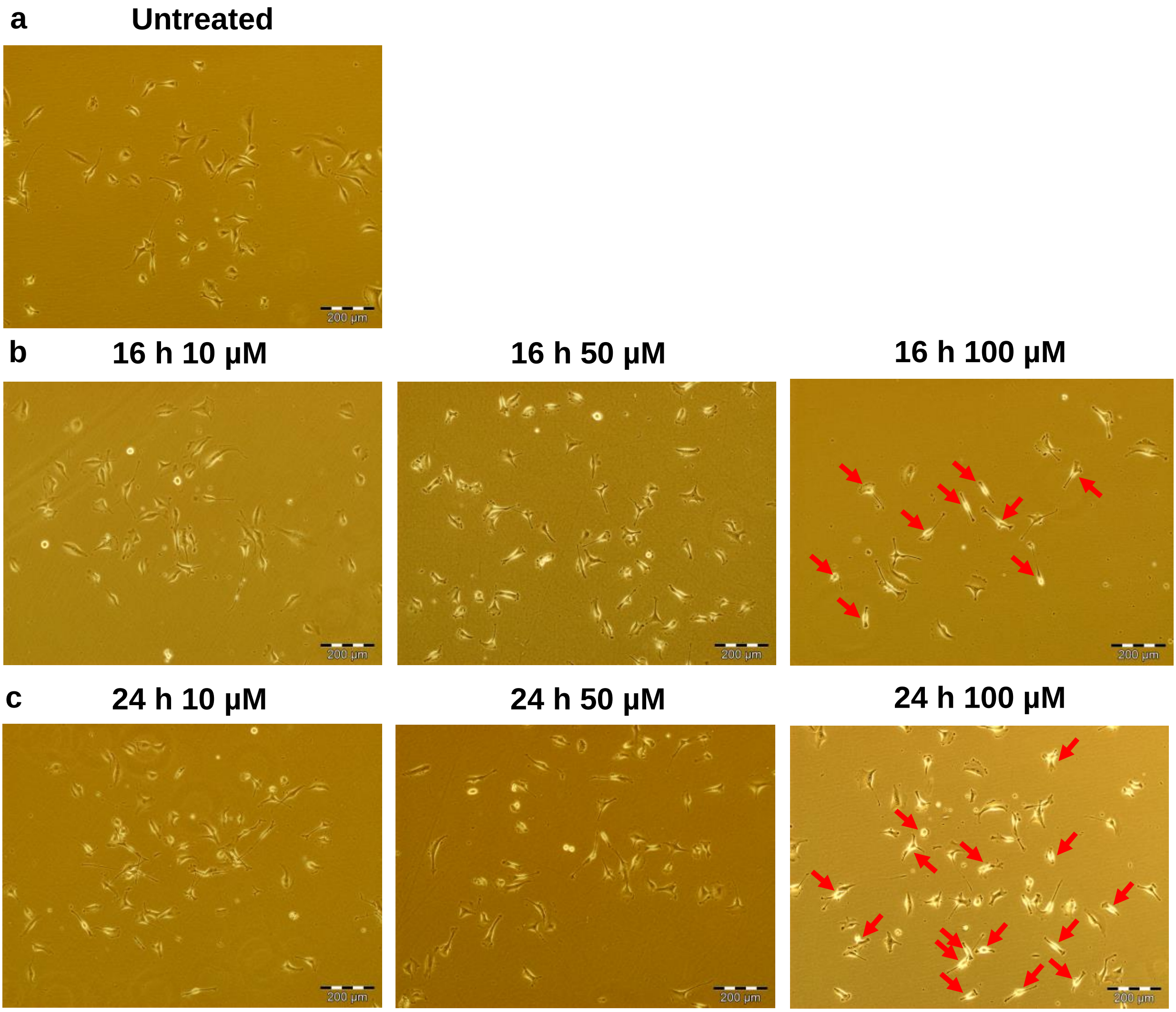

Additional file 3

Microscopic images of NP69 cells after treatment with H_2O_2. Cells were either left untreated (a) or treated with 10, 50 and 100 µM of H_2O_2 for 16 hours (b) and 24 hours (c). Cytoplasmic shrinkage (indicated by the red arrows) was observed in cells treated with 100 µM of H_2O_2 for 16 and 24 hours. Magnification, 100x.
