# Supplementary material for: Oxidative stress-induced chromosome breaks within the ABL gene: a model for chromosome rearrangement in nasopharyngeal carcinoma
Source: Hum Genomics. 2018 Jun 18;12:29. doi: 10.1186/s40246-018-0160-8 (PMC6006577; doi:10.1186/s40246-018-0160-8)
Supplement: Supplementary file 3 — Microscopic images of HK1 cells after treatment with H2O2. (PDF 150 kb) [file 40246_2018_160_MOESM3_ESM.pdf]

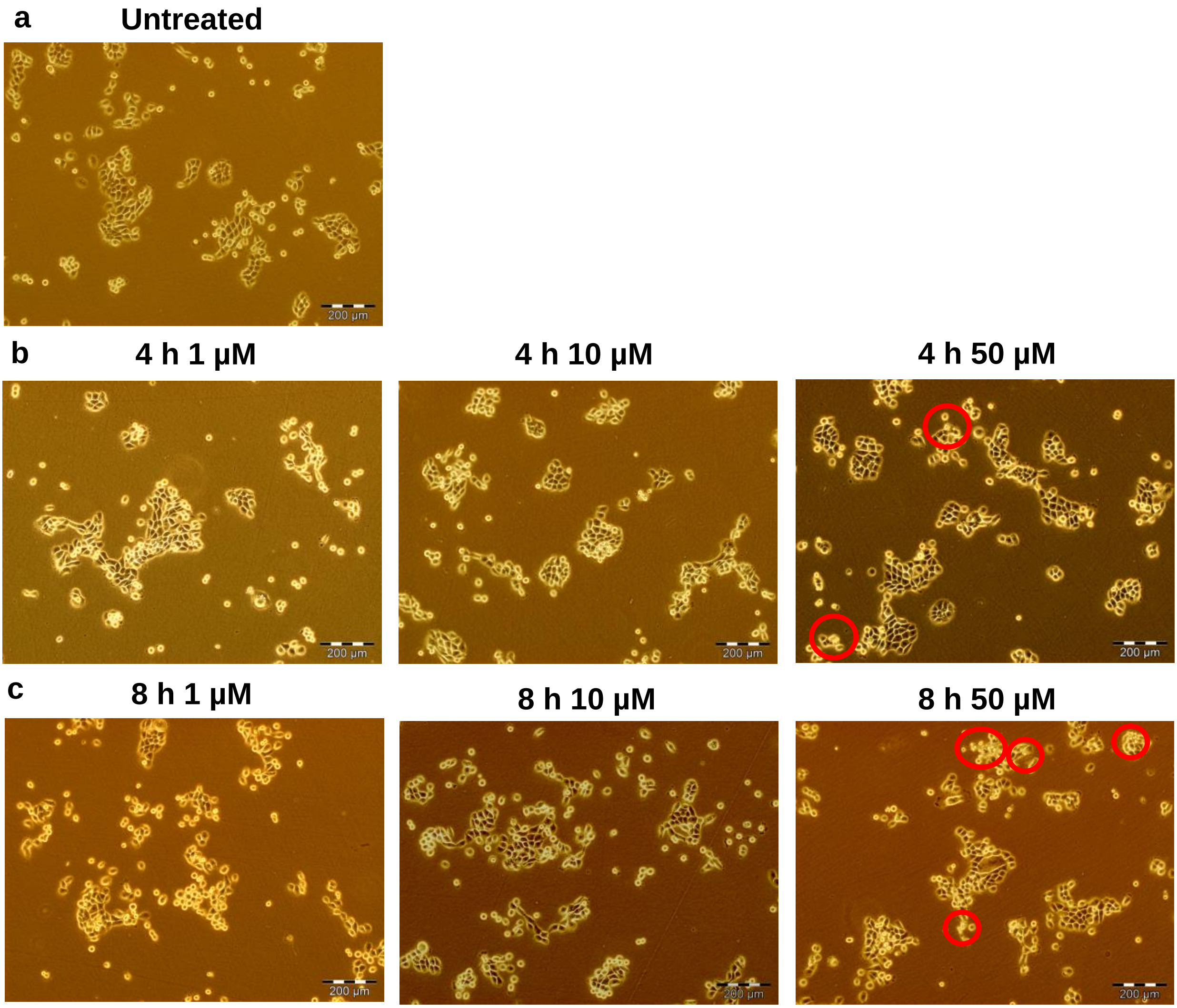

Additional file 4

Microscopic images of HK1 cells after treatment with H_2O_2. Cells were either left untreated (a) or treated with 1, 10 and 50 μM of H_2O_2 for 4 hours (b) and 8 hours (c). Formation of membrane blebbing (indicated by the red circles) was observed in cells treated with 50 μM of H_2O_2 for 4 and 8 hours. Magnification, 100x.
