# Supplementary material for: Oxidative stress-induced chromosome breaks within the ABL gene: a model for chromosome rearrangement in nasopharyngeal carcinoma
Source: Hum Genomics. 2018 Jun 18;12:29. doi: 10.1186/s40246-018-0160-8 (PMC6006577; doi:10.1186/s40246-018-0160-8)
Supplement: Supplementary file 4 — Description of exons and introns in the LHFPL3 gene. (PDF 56 kb) [file 40246_2018_160_MOESM4_ESM.pdf]

**Additional file 2**

**Description of exons and introns in the *LHFPL3* gene**

| *Exon/intron* | *Nucleotide position* | *Length (bp)* |
|---|---|---|
| 5’ upstream sequence | 1-600 | 600 |
| Exon 1 | 601-1169 | 569 |
| Intron 1 | 1170-408618 | 407449 |
| Exon 2 | 408619-408855 | 237 |
| Intron 2 | 408856-578130 | 169275 |
| Exon 3 | 578131-579176 | 1046 |
| 3' downstream sequence | 579177-579776 | 600 |

The *LHFPL3* gene located at 7q22 is 578576 bp in length [Ensembl:ENSG00000187416].
